# Nanobody-Based Blocking of Binding ELISA for the Detection of Anti-NS1 Zika-Virus-Specific Antibodies in Convalescent Patients

**DOI:** 10.3390/tropicalmed8010055

**Published:** 2023-01-10

**Authors:** Triana Delfin-Riela, Martín A. Rossotti, Giada Mattiuzzo, César Echaides, Gualberto González-Sapienza

**Affiliations:** 1 Cátedra de Inmunología, DEPBIO, Facultad de Química, Instituto de Higiene, UDELAR, Montevideo 11600, Uruguay; 2 Division of Virology, National Institute for Biological Standards and Control (NIBSC)-MHRA, Hertfordshire EN6 3QG, UK; 3 Parque Lecoq, IMM, Montevideo 11300, Uruguay

**Keywords:** serology, flavivirus, immunoassay, phage display, single-domain antibodies

## Abstract

Zika virus has spread around the world with rapid pace in the last five years. Although symptoms are typically mild and unspecific, Zika’s major impact occurs during pregnancy, generating a congenital syndrome. Serology plays a key role in its diagnosis. However, its use is limited due to the uncertainty caused by the cross-reaction of antibodies elicited in response to other flavivirus infections when tested in direct immunoassays. Using a panel of previously generated anti-Zika non-structural protein 1 (NS1) nanobodies, a set was selected that only recognizes epitopes present in Zika and is immunogenic to humans. A proper arrangement of these nanobodies was made and conditions were optimized in order to develop a novel serology assay. This new ELISA relies on the inhibition of the binding of a set of selected nanobodies to Zika-immobilized NS1 when previously incubated with Zika convalescent sera. Using the developed blocking of binding assay, it was possible to discriminate between Zika-specific and cross-reactive antibodies in serum samples from infections with Zika and other flaviviruses.

## 1. Introduction

Zika virus (ZIKV) was reported for the first time in Uganda in 1947 and remained almost unnoticed until two major outbreaks took place in French Polynesia and Brazil in 2013 and 2015, respectively [1,2,3,4]. Shortly after that, ZIKV rapidly disseminated and, according to the World Health Organization (WHO), nowadays, it affects more than 80 countries around the globe. ZIKV is an arbovirus from the *Flaviviridae* family and its main route of transmission is by infected *Aedes* spp. mosquito bites. However, it can also spread via sexual contact and is vertically transmitted from the mother to the fetus [5,6]. As a neurotropic virus, it has been associated with *Guillian Barré* Syndrome in adults and during pregnancy with neurological birth defects, collectively known as Congenital Zika Syndrome (CZS) [7]. The method of choice for the diagnosis of acute ZIKV infection is the detection of viral RNA present in the blood by real-time polymerase chain reaction (RT_PCR) [8]. As the viraemia in blood is short-lived (5–7 days), the detection of IgM and IgG antibodies is the most suitable method for diagnosis in the convalescent phase. Due to the high level of similarity among flaviviruses, considerable immunological cross-reactivity has been observed between them [9]. Thus, false-positive anti-ZIKV reactions might be common in regions where Flavivirus infections overlap [10,11]. Flavivirus serology assays are generally based on the detection of antibodies against structural antigens, mostly the envelope protein E. However, structural proteins are highly conserved; thus, the antibodies generated against these proteins present a significant degree of cross-reactivity, leading to a poor diagnosis [11,12]. Solving this problem is critical to count on dependable serological tests for ZIKV infection diagnosis to monitor the general population seroprevalence, especially in pregnant women. Moreover, it is relevant to measure the incidence of CZS among this particular population as well as to identify other possible neurological complications associated with this infection [13]. Therefore, the development of serology methods that could unequivocally diagnose ZIKV in flavivirus-endemic settings is of extreme importance. 

**Figure 1:**
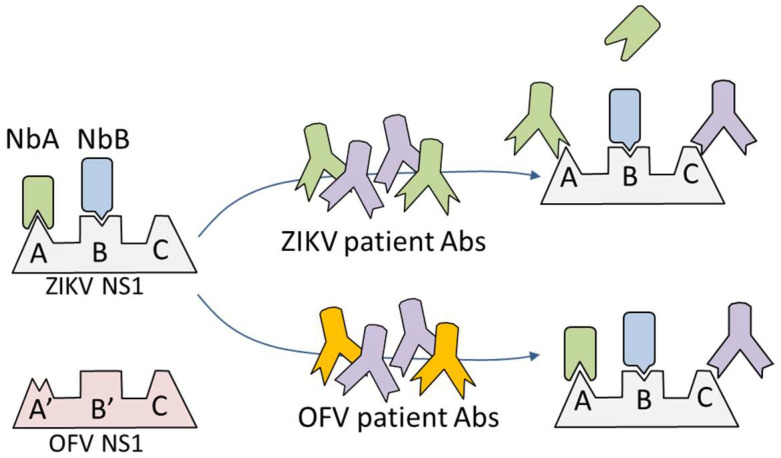
The Non-Structural 1 (NS1) protein from Zika and other flaviviruses (OFV) has some unique epitopes (A, B and A′, B′, respectively) and shares others (represented by C). For the development of a blocking of binding assay, the competing antibodies need to fulfill two conditions: they must react with a ZIKV NS1-specific epitope and that epitope must be immunogenic in human infections with the virus. In the scheme, antibody NbA and NbB do react with unique epitopes, but NbB recognizes an epitope that is not immunogenic in humans and, therefore, there are no blocking antibodies in the serum of the infected patients. Hence, only NbA is suited to develop a blocking of binding assay.

The Non-Structural 1 (NS1) protein is less conserved among flaviviruses; thus, it has been proposed as a more reliable diagnostic biomarker. NS1 induces a strong IgG response and seroconversion occurs as early as 5–8 days after initial symptoms [14,15]. However, despite its lower identity with other viral NS1, several studies have shown that current anti-ZIKV antibody assays using this antigen, while highly sensitive, do not reliably distinguish among flavivirus infections, mostly due to the great extent of cross reactivity observed with serum samples from Dengue virus (DV)-infected patients [16,17]. To overcome this, Balmaseda et al. used a human monoclonal antibody, previously shown to define a specific NS1 ZIKV epitope to develop an inhibition of binding assay, and they could efficiently distinguish ZIKV from other flaviviruses. This ELISA works by comparing the binding of the selective antibody to the immobilized ZIKV NS1 in the presence or absence of infected patient’s sera, as illustrated in Figure 1 [18]. While this represents great progress in the serodiagnosis of Zika infection, a limitation of this assay is the use of a specific anti-ZIKV antibody. The availability of antibodies with similar specificity and known published sequences will make this method reproducible in any laboratory to facilitate and extend the use of this diagnostic format.

The recombinant fragment (nanobodies) derived from the variable domain (VHH) of heavy-chain-only antibodies, found in camelids, has salient biotechnological properties and can be readily reproduced from sequences [19]. These antibodies possess numerous advantages for immunoassay development compared to conventional antibodies, such as high soluble expression levels, small size, excellent thermal stability, simple genetic manipulation, among others [20,21,22]. In the past, we developed high-throughput methods for the generation and selection of nanobodies (Nbs) for sensitive detection of biomarkers in complex matrixes [23,24]. Recently, we developed a large panel of Nbs to ZIKV NS1 and selected pairs of Nbs that allow for the sensitive detection of the antigen in serum samples [25]. In the present study, this panel was further tested to select Nbs defining ZIKV NS1-specific epitopes that can be used in inhibition assays to differentiate ZIKV human infections. Using these antibodies, a blocking of binding ELISA was generated where the binding of nanobodies is strongly inhibited when the antigen is previously incubated with Zika convalescent sera, but not if incubated with other anti-flavivirus sera, thus, allowing for the specific diagnosis of this infection. Furthermore, since these Nbs can be produced recombinantly using the provided sequence, they can be used to develop highly specific, low-cost in-house blocking of binding ELISAs for the reliable detection of ZIKV infections.

## 2. Materials and Methods

### 2.1. Materials

ZIKV NS1 protein as well as NS1 from other flaviviruses, including Dengue 1 (DV1), Yellow Fever (YFV) and Saint Louis (SLV), were purchased from The Native Antigen Company, Inc. (Oxford, UK). ELISA strips and plates (Greiner Bio-One, Monroe, NC, USA). Peroxidase-conjugated streptavidin was obtained from Thermofisher (Rockford, IL, USA). Other conjugated antibodies were acquired from Abcam (Cambridge, MA, USA). 3,3′,5,5′-D-biotin was from Amresco (Wayne, PA, USA). Tetramethylbenzidine (TMB) and other common chemicals were purchased from Sigma-Aldrich (Mississauga, CA, USA).

The WHO 1st international standard (IS) for anti-Asian linage ZIKV antibody (IS 16_352), the Standard Reagents for anti-ZIKV antibody IS 16_320 and sample16_328, convalescent serum pool from recovered ZIKV-infected patients were obtained from the National Institute for Biological Standards and Control, UK. RT-PCR and serology confirmed serum samples from patients infected with different flaviviruses, including ZIKV (n = 3), Dengue (DV, n = 11), Yellow Fever (YFV, n = 8) and Saint Louis (SLV, n = 5), were remnant diagnostic samples provided by the Uruguayan Ministry of Health. Samples from healthy donors from a non-endemic flavivirus zone were available from previous studies. All samples were de-identified and processed following the recommendations of the Comisión de Ética en la Investigación con Seres Humanos of the Facultad de Química, UDELAR.

The nanobodies used in this study have been extensively described by Delfin-Riela et al. 2020 and their amino acid sequences are published [25].

### 2.2. Evaluation of Direct ELISA for Antibodies Detection of Flavivirus Immune Sera

ELISA plates were coated with 100 µL (200 ng/mL) of ZIKV NS1 overnight (ON) at 4 °C. Plates were washed with PBS-0.05% Tween 20 (PBS-T) and blocked with PBS-1% bovine serum albumin (BSA) for 1 h (1 h) at room temperature (RT). After washing, each plate was incubated for 1 h with the flavivirus, 1/500 diluted, immune serum. A set of naïve sera was also included as a negative control. After washing, 100 µL of a mix of anti-IgG:HRP and anti-IgM:HRP at manufacturer’s recommended concentration was loaded and incubated for 1 h at RT. Finally, plates were washed with PBS-T, and TMB substrate was added for ten minutes. The enzyme reaction was stopped by addition of 50 μL of 2N H_2_SO_4_ and the optical density (OD) was then measured at 450 nm with a Fluostar Optima Reader (BMG, Ortenberg, Germany).

### 2.3. Selection of Nanobodies: Binding Inhibition and Cross-Reactivity Evaluation

High-binding polystyrene plates were coated with 200 ng/mL of ZIKV NS1 in PBS, ON at 4 °C. Plates were blocked with PBS-1% BSA for 1h at RT and then washed with PBS-T. One hundred microliters of ZIKV serum standards diluted with PBS (1/20, 1/40 for 16-352, and 1/80 for 16-320 and 16-328) or PBS (blank) was added and incubated for 1 h at RT. After washing with PBS-T, the plates were loaded with 100 µL of 10 biotinylated anti-NS1 Nbs for 1 h at RT. The Nb concentrations (5.0 to 12 ng/mL) were previously selected as the amount of Nb capable of generating an OD 450 nm of 1UA when exposed to ZIKV NS1. Plates were washed with PBS-T, and peroxidase-conjugated streptavidin was added and incubated for 1 h at RT. Finally, plates were washed with PBS-T and TMB substrate was added for ten minutes. The enzyme reaction was stopped by addition of 50 μL of 2N H_2_SO_4_ and OD 450 nm was then measured. The percentage of Binding Inhibition (%BI) was calculated as follows: %BI = [1 − (OD sample/OD PBS)] × 100, where OD PBS is the absorbance of the blank.

For the cross-reactivity study, the procedure was identical to that described above but using flavivirus convalescent serum pools instead of the anti-ZIKV standard. The obtained %BI was compared to that attained with the IS 16_352 standard.

### 2.4. Optimization of the ELISA

High-binding polystyrene plates were coated with 200 ng/mL of ZIKV NS1 in PBS, ON at 4 °C. Plates were blocked with PBS-1% BSA for 1 h at RT and then washed with PBS-T. Hundred µL of serial dilutions, starting from 1/20 of the three standard sera, was incubated for 1 h at RT. After washing with PBS-T, the pool of Nbs (consisting of equal amounts of each antibody) was added and incubated for an additional 1 h at RT. Plates were washed with PBS-T and Stp-HRP was added and incubated for 1 h at RT. Finally, the reaction was developed as mentioned above. The working dilution was defined as the one that produces a high %BI (over 50%) and does not show any inhibition with negative samples. Using a fixed dilution (1/40) of standards 16-328 and 16-352, a variant of the assay was performed. In this case, the incubation of the pool of sera and the Nbs was performed simultaneously. The remaining steps were equally performed as described above.

The cut-off point of the assay was estimated as the mean %BI obtained from 98 ZIKV-negative samples tested in the Nb-BI ELISA, plus 3 standard deviations.

Cross-reactivity of the Inhibition ELISA was determined using individual flavivirus immune serum samples from patients infected with DV, SLV and YFV. Individual samples from ZIKV-positive patients and anti-ZIKV standard sera were also performed in the same conditions as mentioned above. The %BI achieved by ZIKV-positive and ZIKV-negative samples was compared to the cut-off value.

### 2.5. Study of Intra-Assay and Inter-Assay Reproducibility

The precision of the method was evaluated by calculating the coefficient of variation (CV) using the IS 16_352 standard, processed at a dilution of 1/40. Intra-assay variation was calculated by making five replicates of the standard in the same day. The inter-assay variability was calculated by testing the standard in quintuplicates, during five consecutive days.

## 3. Results

### 3.1. Election of Nanobodies: Binding Inhibition and Cross-Reactivity Evaluation

**Figure 2:**
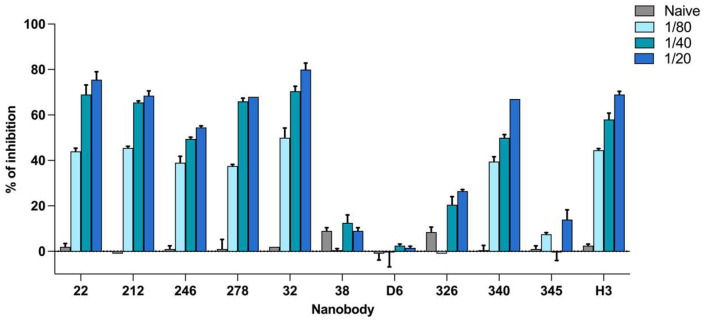
Inhibition achieved over different nanobodies by anti-Zika standard serum. Nanobodies were challenged with three dilutions of WHO IS 16_352 standard and with a pool of naïve sera (1/20 diluted). The percentage of binding inhibition for each Nb is shown. Measurements are the average of duplicates.

In spite of the fact that the NS1 proteins are less conserved among flaviviruses than structural proteins, there is a large extent of cross-reactivity when a convalescent serum infected with a non-ZIKV flavivirus is tested directly against ZIKV NS1. This behavior was demonstrated by the evaluation of the reactivity of anti-DV, anti-YF and anti-SLV serum against ZIKV NS1 immobilized on the solid phase (see Supplementary Figure S1). This is mainly due to the ability of antibodies to recognize conserved epitopes of the NS1 shared among flaviviruses. One possibility to overcome this drawback is to devise a blocking of binding test. As shown by Balmaseda et al., this can be carried out by studying the binding inhibition (%BI) of a monoclonal antibody that selectively targets ZIKV epitopes, in the presence or absence of the patient serum [18]. We recently generated a large panel of Nbs against ZIKV NS1 with negligible cross-reactivity for DV1, SLV, YFV and West Nile virus [25]. Eleven of these Nbs (22, 212, 246, 278, 32, 38, 326, 340, 345, D6 and H3) were chosen with the purpose of developing a blocking of binding test. To this aim, in the first place, Nbs were further selected according to their capability of being inhibited by human anti-ZIKV antibodies. This was tested using the WHO anti-ZIKV standard serum IS 16_352. Previously, the Nbs were titrated against ZIKV NS1 protein and an optimal concentration corresponding to an optical density (OD) of 1.0 UA was selected for each of them (data not shown). The extent of binding inhibition caused by the standard serum in these conditions is shown in Figure 2. As can be observed, the Nbs 38, D6, 326 and 345 were not inhibited by the reference serum. Considering that this standard consists of a pool of several human sera, it may indicate that these Nbs define epitopes that are not immunogenic in human ZIKV infections and, therefore, were excluded. Conversely, the rest of the Nbs showed a relevant degree of inhibition, up to 80% at the highest standard concentration (1/20). To test if this response is affected by the particular immune response of the patients included in IS 16_352, two different anti ZIKV NS1 reference sera were assayed in a similar experiment and no significant difference was found (see Supplementary Figure S2). This fact implies that the selected Nbs were inhibited by anti-ZIKV serum samples with different characteristics, such as region and time of infection, among others.

**Figure 3:**
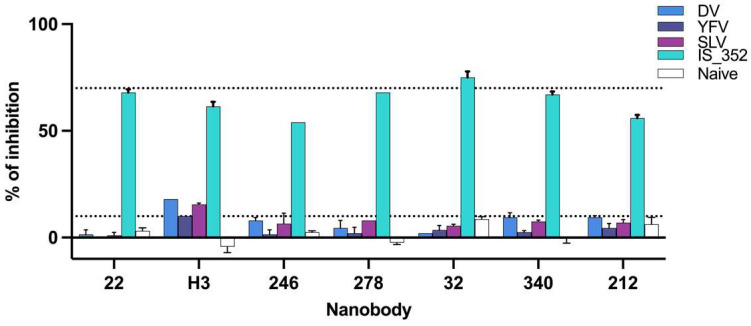
Cross-reactive nanobody inhibition by flavivirus immune sera. Percentages of inhibition achieved for each nanobody by immune serum pools of: Dengue Virus (DV), Yellow Fever Virus (YFV) and Saint Louis Virus (SLV), compared to those generated by WHO IS 16_352 standard and naïve serum. Dotted lines correspond to 10% and 70% of binding inhibition. Measurements are the average of duplicates.

Next, we tested whether the antibodies present in the serum of patients infected with other flaviviruses were able to inhibit the binding of the selected Nbs. To this end, ZIKV NS1 was incubated with various flavivirus convalescent serum pools and naïve serum (Figure S3). The %BI for each Nb was calculated and compared to that obtained with the anti-ZIKV standard IS 16_352, Figure 3. As observed, NbH3 showed a moderate cross-reactivity with other flavivirus serum pools. This Nb had to be discarded because, despite lacking reactivity against DV1, WNV, YFV and SLV NS1, it seems to define an epitope that partially overlaps with the region of ZIKV NS1 recognized by antibodies present in patients affected by other flavivirus infections.

Next, we studied whether the remaining Nbs, 22, 246, 278, 32, 340 and 212, defined unique or overlapping epitopes. No complementarity was found for any of their all-against-all pairwise combinations in two-site ELISA (data not shown). Considering that there are considerable differences in their complementary-determining region (CDR) sequences, most probably, they react with largely overlapping but not identical epitopes (see Supplementary Figure S4). Moreover, these Nbs appear to target a region of ZIKV NS1 that is highly immunogenic, both in the immunized llama and ZIKV patients, but does not lead to the generation of cross-reactive antibodies in infections with other flaviviruses. For this reason, we decided to set up our inhibition test using a pool of these six Nbs (consisting of equal amounts of each Nb).

### 3.2. Optimization of Inhibition ELISA

**Figure 4:**
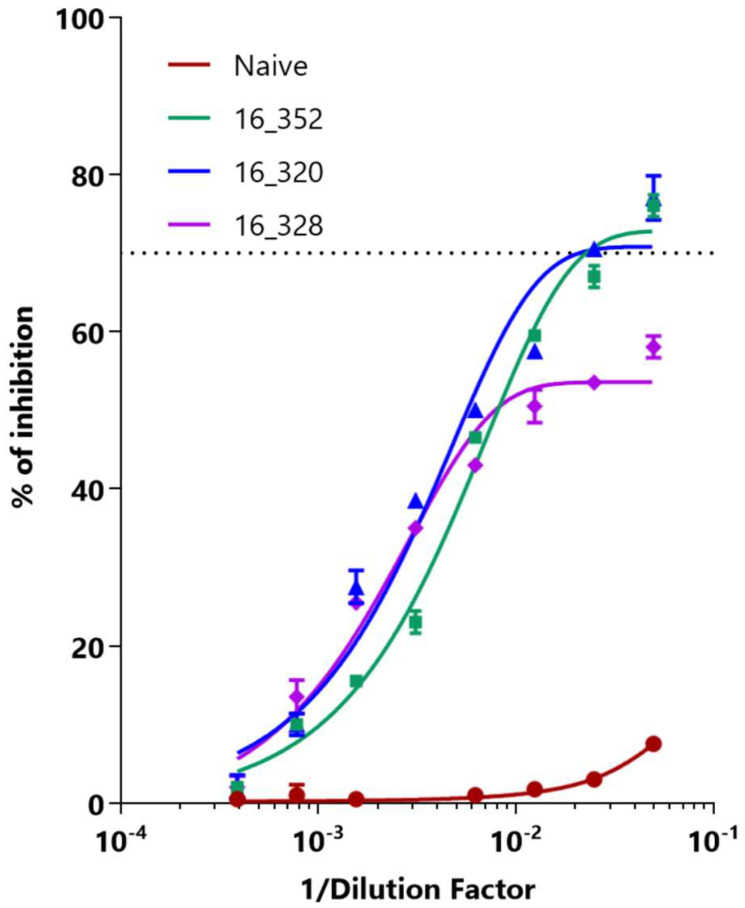
**Selection of sample working dilution from standard curves**. Dilutions from 1/20 to 1/2560 of standards as well as a pool of naïve sera (circles) were analyzed. Measurements were made in duplicates. 16_352 (squares), 16_320 (diamonds) and 16_328 correspond to WHO International Standards.

To determine the appropriate dilution to process the samples, inhibition curves were performed using the three anti-ZIKV standards, Figure 4. As observed, even with a 1/1280 dilution, there was detectable inhibition for all three WHO international standards. Considering that no significant inhibition was observed with the pool of negative sera, we selected 1/40 as working dilution in order to maximize the specificity. Once an appropriate dilution was set, aiming to maximize the inhibition effect, we analyzed the effect of the order in which serum and Nbs were added. Sequential and simultaneous additions were compared, using two different anti-ZIKV standards. As expected, pre-incubation of the immobilized antigen with the patient serum achieved a significant improvement in the %BI, about 20%, compared to the simultaneous incubation of the patient serum and nanobodies, avoiding competition (see Supplementary Figure S5).

**Figure 5:**
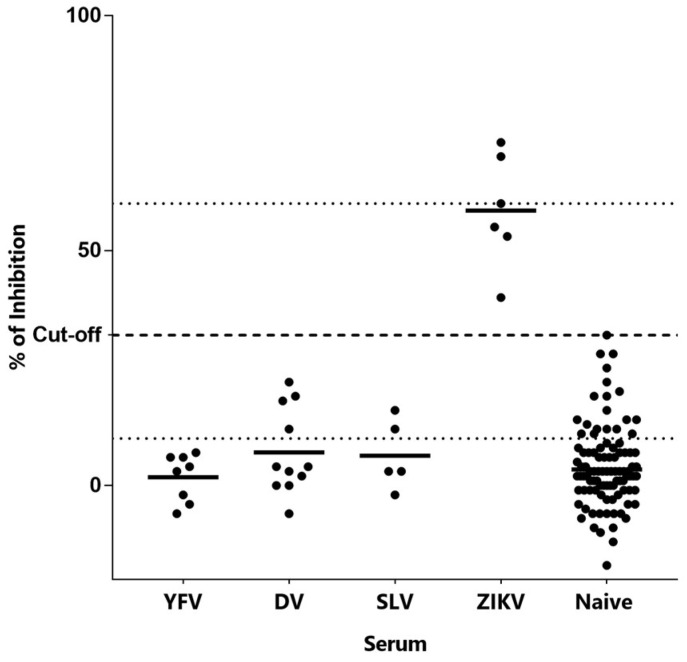
Inhibition achieved by flavivirus convalescent sera. Samples of Yellow Fever Virus (YFV), Dengue Virus (DV), Saint Louis Virus (SLV) and Zika Virus (ZIKV) as well as naïve sera were examined in duplicates.

Once the conditions to perform the test were established, an available panel of anti-ZIKV and other flavivirus convalescent sera was used to evaluate its diagnostic potential, Figure 5. First of all, an estimation of a cut-off value was calculated as the average inhibition percentage of 98 negative sera plus three standard deviations, resulting in 32%. By using this cut-off value, all non-Zika samples (n = 24) and 97/98 true-negative sera were classified as negatives. Likewise, the six ZIKV true-positive sera tested were also correctly classified as positive. Among these, it is interesting to highlight that one of the ZIKV-positive samples, which was collected barely 10 days after symptom onset when antibodies are just starting to appear, was also above the cut-off value. Hence, the developed assay, by introducing a set of competitive Nbs selective for a human-immunogenic ZIKV NS1 epitope, was able to overcome the cross-reactivity limitations faced by the direct antibody-detection approach (compare to Figure S1). This is especially relevant in areas where more than one virus of this family co-circulates, where the antibodies generated by a past infection against another flavivirus could recognize a ZIKV NS1.

### 3.3. Study of the Intra-Assay and Inter-Assay Reproducibility

The precision of the method was evaluated by testing the IS 16_352 NIBSC standard in quintuplicates on the same day (intra-day precision) and five different days (inter-day precision), Table 1. In both cases, the coefficient of variation in percentage (CV%) was in accordance with the acceptance criteria (CV < 20%) recommended for clinical assays [26].

## 4. Conclusions

The development of antibody-detection methods that could unequivocally diagnose ZIKV in flavivirus-endemic settings is of extreme importance. Blocking of binding tests represent major progress in the diagnosis of flavivirus disease because they overcome the limitation caused by the presence of cross-reactive antibodies commonly detected by direct tests. The aim of this study was to identify nanobodies to ZIKV-specific epitopes that could be used to produce a blocking of binding test. As starting point, a panel of ten ZIKV NS1-specific nanobodies was used, but interestingly, not all of them turned out to be useful. Indeed, the binding of four of these clones was not blocked by the antibodies present in ZIKV patient sera, which demonstrates that the antibodies used as probes for diagnostic inhibition tests should address epitopes that are immunogenic in ZIKV infections. In addition, another Nb had to be discarded because, despite lacking reactivity against DV1, WNV, YFV and SLV NS1, it seems to define an epitope that partially overlaps with the region of ZIKV NS1 recognized by antibodies present in patients affected by other flavivirus infections. Finally, the remaining six nanobodies that differ strongly in their CDR sequences appear to target a region of ZIKV NS1 that was highly immunogenic, both in the immunized llama and, more importantly, in ZIKV patients, but which is not recognized by antibodies raised by infections with other flaviviruses. Although, a large serum panel was not available at the time of this study, using the selected nanobodies, it was possible to set up a sensitive test that specifically discriminates ZIKV from other flavivirus infections in an unequivocal manner. In addition, given that nanobodies are perpetuated in silico, laboratories can reproduce them from their sequence in an affordable and standardized fashion, opening the opportunity for local developments, which, after further validation, will help to build diagnostic capacity for this infection, even in low-resource settings.

## Figures and Tables

**Table 1:** Precision parameters.

	Intra-Day Precision	Inter-Day Precision
Replicates	5	5
Mean Value (% inhibition)	68	65
CV%	2.7	7.7

NIBSC standard IS 16-352 was used at a dilution of 1/40.

## Data Availability

Not applicable.

## Supplementary Materials

The following supporting information can be downloaded at: https://www.mdpi.com/article/10.3390/tropicalmed8010055/s1, Figure S1. Reactivity of serum IgG present in DV, YFV, SLV and ZIKV immune patients with ZIKV NS1; Figure S2. Inhibition test performed with individual nanobodies using three standard sera; Figure S3. Cross reactive nanobody-inhibition by Flavivirus immune sera; Figure S4. Sequence alignment of the Nbs used for binding of inhibition tests; Figure S5. Inhibition test performed sequentially or simultaneously.
