# Supplementary material for: Nanobody-Based Blocking of Binding ELISA for the Detection of Anti-NS1 Zika-Virus-Specific Antibodies in Convalescent Patients
Source: Trop Med Infect Dis. 2023 Jan 10;8(1):55. doi: 10.3390/tropicalmed8010055 (PMC9862682; doi:10.3390/tropicalmed8010055)
Supplement: Supplementary file 1 [file tropicalmed-08-00055-s001.zip › tropicalmed-2119229-supplementary.pdf]

Supplementary Material

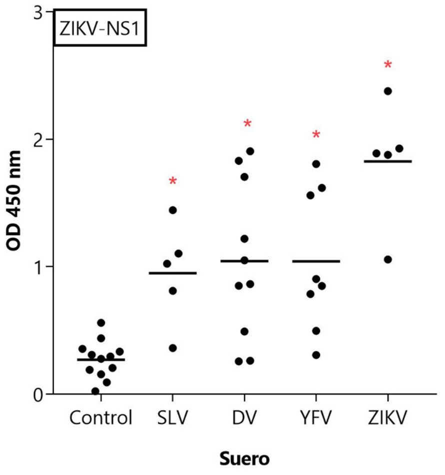

Figure S1. Reactivity of serum IgG present in DV, YFV, SLV and ZIKV immune patients against ZIKV NS1. Sera were tested at 1/100 dilution for binding of total Ig to wells coated with ZIKV NS1. Sera: DV (n=10), YFV (n=8), SLV (n=5) and ZIKV (n=6). Mann-Whitney test was applied for the significance analysis

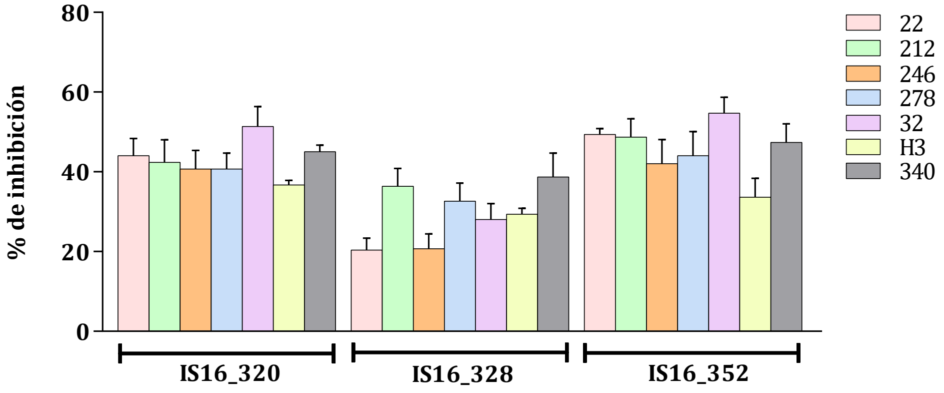

Figure S2. Inhibition test performed with individual nanobodies using three standard serums. All sera were tested in 1/80 dilutions.

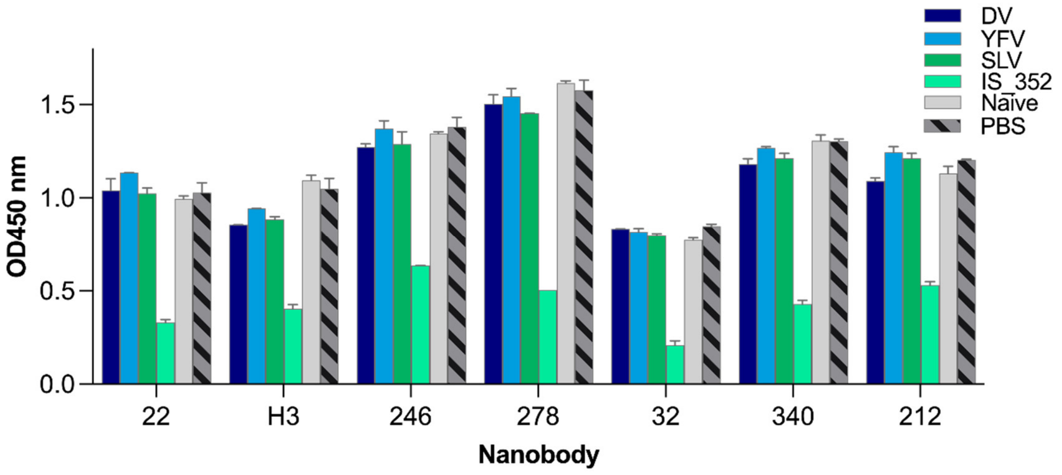

Figure S3. Cross reactive nanobody-inhibition by Flavivirus immune sera. Optical density achieved for each nanobody by immune serum pools of: Dengue Virus (DV), Yellow Fever Virus (YFV) and Saint Louis Virus (SLV), compared to those generated by WHO IS 16_352 standard, naïve serum and PBS. Dotted lines correspond to 10% and 70% of binding inhibition. Measurements are the average of duplicates.

```
    ---------FR1------------- --CDR1--- ------FR2-------  -CDR2--

22  EVQLVESGGGLVQTGGSLRLSCAAS GTIFSTKA- MGWYRQAPGKRREFVAL IAPGGDI

278 QVQLVQSGGGLVRPGGSLRLSCAAS GNIFSTKA- MGWYRQAPGKRREFVAL IDPAGST

340 QVKLVESGGGLVQPGGSLRLSCAVS GTFSSITS- MGWYRQAPGKQRELVAT FSGGRTN

212 QVQLVQSGGGLVQAGGSLRLSCAAS GNIFSSNAV GWWRRAPGRQQREWVAT ITSGDST

32  EVQLVESGGGLVQAGGSLRLSCAVS GIDFSRYAI TWNRQSPGNQRREWVAT LPPADTT

246 EVQLVESGGGRVQAGGSLRLSCAGS ARLSSIKA- MQWSRQAPGKQREWVAT VTPGGST


    -----------------FR3------------------- ---CDR3------ ----FR4----

22  TYADSAEGRFTISRDSAKGTV-WLQMNDLKAEDTAVYYC NTVPRTQD----- WGQGTQVTVSS

278 TYADSVEGRFTISKDSAKGTV-WLQMNDLKAEDTAVYYC NTVPRVQD----- WGQGTQVTVSS

340 YVDSVKGRFTVSRDNARSTVDLYLQMNSLKPEDTAVYYC NVEGLWNNRRGRA WGQGTQVTVSS

212 HYADSVEGRFTISGDNAKNTV-YLQMDSLKPEDTAVYYC TTVPRRGD----- WGQGTQVTVSS

32  VYADAVKGRFTISRDNTKNTV-YLQMNSLKPEDTAVYYC ATSPRIHN----- WGQGTQVTVSS

246 IYADSVEGRFTISRDNAKNTV-YLQMDNLKPEDTGMYYC NEMPRIMP----- WGQGTQVTVSS
```

Figure S4. Sequence alignment of the Nbs used for binding of inhibition tests. The regions corresponding to the frameworks (FR) and the three CDRs are denoted on top. Gaps are shown as dashes.

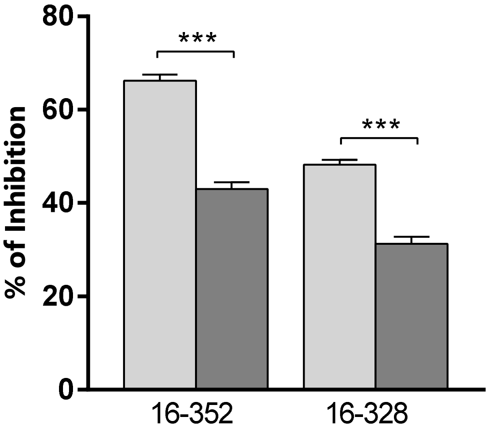

Figure S5. Inhibition test performed sequentially (light gray) or simultaneously (gray). Two conditions were used: a) Standard serum was added first, and after washing the plate was incubated with the competing nanobodies (light gray), and b) standard serum and the competing nanobodies were added simultaneously (gray). Standard sera 16-352 and 16-328 were tested 1/40 dilution. ANOVA one way test was applied for the significance analysis.
